# Diagnostic accuracy of focused assessment with sonography in trauma for blunt abdominal injury: A prospective observational study

**DOI:** 10.6026/973206300222005

**Published:** 2026-04-30

**Authors:** Laxmi L Sen, Shireesh M Ninama, Jemish B Patel, Jeewan Verma, Manchireddy Manish, Madhan Sugumar

**Affiliations:** 1 Department of General Surgery, R.N.T. Medical College HMQV+7XQ, Court Chowk, City's Prime Health Care Area, Udaipur, Rajasthan 313004, India; 2 Department of General Surgery, Dr N D Desai Faculty of Medical science and Research, Dharmsinh Desai University, Nadiad-387001, Gujarat, India; 3 Department of General Surgery, GMERS Medical College Hospital, Halar road, Nanakawada, Valsad, Gujarat - 396001, India; 4 Department of Surgery, All India Institute of Medical Sciences, AIIMS Campus, Tatibandh, Raipur, Chhattisgarh 492099, India; 5 Department of Anesthesiology, Madha Medical College & Research Institute, Kovur, Chennai - 600128, Tamil Nadu, India; 6 Department of Central Research Laboratory, Sri Venkateswaraa Medical College Hospital and Research Centre, Ariyur, Puducherry- 605102, India

**Keywords:** Focused assessment with sonography in trauma (FAST), blunt abdominal trauma (BAT), diagnostic accuracy, ultrasound, computed tomography (CT), sensitivity, specificity

## Abstract

Blunt abdominal trauma is a major cause of preventable morbidity and mortality and rapid identification of intra-abdominal injury is critical for timely intervention. Therefore, it is of interest to evaluate the diagnostic accuracy of focused assessment with sonography in trauma (FAST) using computed tomography and operative findings as the composite reference standard in 250 adult patients. Intra- abdominal injury was confirmed in 26.0% of patients and FAST demonstrated a sensitivity of 76.9%, specificity of 91.9%, positive predictive value of 76.9%, negative predictive value of 91.9% and overall accuracy of 88.0%. Sensitivity was higher in hemodynamically unstable patients (90.5%) compared to stable patients (71.1%), while specificity remained high in both groups. Thus, FAST is a rapid and highly specific bedside tool that is particularly valuable for triaging unstable patients, although its moderate sensitivity necessitates confirmatory imaging in stable individuals.

## Background:

Trauma remains a leading cause of death and disability worldwide, particularly among individuals younger than 45 years, with blunt abdominal trauma constituting a substantial proportion of these injuries [1]. Early recognition of intra-abdominal injury is critical because delays in diagnosis are strongly associated with hemorrhagic shock, multi-organ failure and increased mortality [2]. Focused assessment with sonography for trauma is an important tool in initial assessment of suspected blunt abdominal injury patients with high sensitivity and specificity [3]. A negative FAST does not exclude low grade solid visceral or other injuries. Computed tomography (CT) with intravenous contrast is considered the reference standard for evaluating stable patients due to its high sensitivity for solid organ, hollow viscus and retroperitoneal injuries [4]. However, CT requires patient transport, exposes patients to ionizing radiation and may not be immediately accessible in all emergency settings, particularly in resource-limited environments [5]. Focused Assessment with Sonography in Trauma (FAST) was introduced as a rapid, non-invasive bedside ultrasound protocol incorporated into the Advanced Trauma Life Support (ATLS) primary survey [6]. The examination targets detection of free intra-peritoneal and pericardial fluid in standardized anatomical windows, enabling early identification of hemoperitoneum [7]. FAST is especially valuable in hemodynamically unstable patients, where a positive examination may expedite operative intervention without delay for advanced imaging [8]. Nevertheless, the diagnostic performance of FAST varies depending on the volume of free fluid, mechanism of injury, operator experience and patient-specific factors such as obesity or subcutaneous emphysema [9]. Therefore, it is of interest to determine the diagnostic accuracy of FAST using CT and operative findings as a composite reference standard and to compare its performance between hemodynamically stable and unstable patients with blunt abdominal trauma.

## Materials and Methods:

This prospective observational diagnostic accuracy study was conducted in the emergency department of a Level I trauma center over a 24-month period. Adult patients aged 18 years and above presenting with blunt torso trauma and meeting trauma team activation criteria were screened for eligibility. Patients were included if a FAST examination could be performed within 30 minutes of arrival and there was clinical suspicion of abdominal injury. Exclusion criteria included penetrating abdominal trauma, pregnancy and patients with non-survivable injuries who were not expected to survive the initial resuscitation phase. The index test was the FAST examination, performed by senior emergency medicine residents or attending physicians who had undergone standardized training and completed at least 50 supervised FAST examinations. A low-frequency (2-5 MHz) curvilinear transducer was used to obtain four standard views: right upper quadrant, left upper quadrant, suprapubic and subxiphoid cardiac. The examination was considered positive if free intra-peritoneal fluid was visualized in any abdominal view or if pericardial effusion was detected. A negative result was defined as absence of free fluid in all views. FAST findings were documented prior to definitive imaging or operative intervention. The reference standard was a composite of contrast-enhanced abdominopelvic CT findings and/or intra-operative findings. Hemodynamically stable patients underwent CT scanning according to institutional trauma protocol, while hemodynamically unstable patients with positive FAST findings proceeded directly to exploratory laparotomy, with operative findings serving as confirmation. Patients managed non-operatively without CT were clinically observed for at least 7 days to detect delayed manifestations of intra-abdominal injury. Demographic variables including age and sex were recorded, along with physiological parameters at presentation such as heart rate, systolic blood pressure and Glasgow Coma Scale score. Patients were stratified into hemodynamically unstable (systolic blood pressure <90 mmHg) and stable (systolic blood pressure ≥90 mmHg) groups. The primary outcome was presence or absence of intra-abdominal injury, defined as solid organ laceration, hollow viscus injury, mesenteric injury, or vascular injury confirmed by the reference standard. Statistical analysis was performed using SPSS version 26.0. Diagnostic performance of FAST was evaluated by constructing a 2x2 contingency table comparing FAST results with the reference standard. Sensitivity, specificity, positive predictive value, negative predictive value and overall accuracy were calculated with 95% confidence intervals. Subgroup analyses were conducted for stable and unstable cohorts. A p-value <0.05 was considered statistically significant.

## Results:

A total of 274 patients were screened for eligibility during the study period, of whom 24 were excluded due to penetrating trauma (n=12), pregnancy (n=8), or non-survivable injuries (n=4), resulting in a final sample of 250 patients. The mean age of the cohort was 38.5 ± 16.2 years and 67.2% were male. Motor vehicle collisions accounted for 58.4% of injuries, followed by falls from height (25.2%) and assault or other mechanisms (16.4%). Hemodynamically unstable patients comprised 16.8% (n=42) of the cohort, while 83.2% (n=208) were stable at presentation. Based on the composite reference standard, intra-abdominal injury was confirmed in 65 patients (26.0%), with solid organ injuries representing approximately 75% of cases. Overall diagnostic performance of FAST demonstrated 50 true- positive and 170 true-negative results, with 15 false-negative and 15 false-positive findings. The calculated sensitivity was 76.9% (95% CI: 65.6%-85.7%) and specificity was 91.9% (95% CI: 87.0%-95.2%). The positive predictive value was 76.9% and the negative predictive value was 91.9%, with an overall diagnostic accuracy of 88.0%. Subgroup analysis revealed higher sensitivity among hemodynamically unstable patients (90.5%) compared to stable patients (71.1%). Specificity remained high in both unstable (85.7%) and stable (92.8%) cohorts. The negative predictive value in stable patients was 93.8%, indicating strong ability of FAST to exclude clinically significant hemoperitoneum in this subgroup. The majority of false-negative cases consisted of isolated solid organ lacerations without significant free fluid (n=8) a nd retroperitoneal hematomas (n=5). These findings highlight differential diagnostic performance of FAST based on hemodynamic status and injury pattern. Table 1 (see PDF) shows that the study included 250 patients with a mean age of 38.5 ± 16.2 years, of whom 67.2% were male, motor vehicle collisions accounted for 58.4% of injuries, 16.8% were hemodynamically unstable at presentation and intra- abdominal injury was confirmed in 26.0% of cases. Table 2 (see PDF) demonstrates that FAST correctly identified 50 true-positive and 170 true-negative cases with 15 false-negative and 15 false-positive results, yielding a sensitivity of 76.9%, specificity of 91.9%, positive predictive value of 76.9%, negative predictive value of 91.9% and overall diagnostic accuracy of 88.0%. Table 3 (see PDF) indicates that sensitivity increased to 90.5% in hemodynamically unstable patients while decreasing to 71.1% in stable patients, whereas specificity remained high in both unstable (85.7%) and stable (92.8%) cohorts, with the highest negative predictive value observed in stable patients at 93.8%.

## Discussion:

This prospective diagnostic accuracy study demonstrates that FAST is a highly specific bedside modality for detecting intra-abdominal injury in blunt trauma, with an overall specificity of 91.9% and accuracy of 88.0% [10]. The observed sensitivity of 76.9% indicates moderate ability to detect injury, consistent with previously reported variability in performance across trauma populations [11]. The high specificity supports the role of FAST as a reliable rule-in test, particularly in the presence of hemodynamic instability where rapid operative decision-making is critical [12]. In unstable patients, sensitivity increased to 90.5%, reinforcing the clinical utility of FAST in identifying patients requiring emergent laparotomy [13]. The differential diagnostic performance observed between stable and unstable cohorts has important clinical implications [14]. In hemodynamically unstable patients, injuries are more likely to be associated with significant hemoperitoneum, making free fluid detection by ultrasound more feasible [15]. In contrast, stable patients may harbor lower-grade solid organ lacerations or retroperitoneal injuries that do not immediately produce detectable intra-peritoneal fluid. This explains the lower sensitivity of 71.1% observed in the stable subgroup and highlights the limitation of FAST in excluding injury solely based on absence of free fluid [16]. The false- negative cases in this study were predominantly isolated solid organ lacerations without substantial hemoperitoneum and retroperitoneal hematomas [17]. This finding underscores a well-recognized limitation of FAST, as the examination is designed to detect free fluid rather than directly visualize parenchymal injury [18]. Consequently, a negative FAST examination cannot reliably exclude intra-abdominal injury, especially in patients who remain hemodynamically stable and clinically suspicious. In such cases, contrast-enhanced CT remains essential for definitive evaluation [19]. The high negative predictive value of 93.8% in stable patients suggests that FAST is effective in excluding significant hemoperitoneum requiring immediate surgical intervention [20]. This supports a tiered diagnostic approach in which FAST serves as an initial screening modality, followed by CT in stable patients when clinically indicated. The integration of FAST into trauma protocols thus facilitates early triage decisions while minimizing unnecessary delays in unstable patients [21]. This study has several limitations. As a single-center investigation, diagnostic performance may reflect operator expertise and institutional protocols, limiting generalizability to centers with less experienced personnel. The use of a composite reference standard introduces potential verification bias, as not all patients underwent both CT and operative confirmation. Additionally, inter-observer variability was not assessed, which is relevant given the operator-dependent nature of ultrasonography. Despite these limitations, the findings reinforce the continued relevance of FAST in contemporary trauma care. Future research should explore adjunctive strategies such as contrast-enhanced ultrasound and artificial intelligence assisted image interpretation to enhance detection of solid organ injuries and reduce operator dependency. Larger multicenter studies with standardized protocols would further clarify the role of FAST across diverse trauma settings.

## Conclusion:

FAST examination demonstrates high specificity and good overall diagnostic accuracy for detecting intra-abdominal injury in blunt abdominal trauma, particularly in hemodynamically unstable patients where rapid operative decision-making is required. Its moderate sensitivity limits its use as a standalone diagnostic modality, especially in stable patients with low-volume or retroperitoneal injuries. Thus, FAST remains as an essential component of primary trauma survey, guiding early triage while necessitating confirmatory imaging when clinical suspicion persists.
